# Text mining meets community curation: a newly designed curation platform to improve author experience and participation at WormBase

**DOI:** 10.1093/database/baaa006

**Published:** 2020-03-17

**Authors:** Valerio Arnaboldi, Daniela Raciti, Kimberly Van Auken, Juancarlos N Chan, Hans-Michael Müller, Paul W Sternberg

**Affiliations:** Division of Biology and Biological Engineering 156–29, California Institute of Technology, 1200 E California Blvd, Pasadena, CA 91125, USA

## Abstract

Biological knowledgebases rely on expert biocuration of the research literature to maintain up-to-date collections of data organized in machine-readable form. To enter information into knowledgebases, curators need to follow three steps: (i) identify papers containing relevant data, a process called triaging; (ii) recognize named entities; and (iii) extract and curate data in accordance with the underlying data models. WormBase (WB), the authoritative repository for research data on *Caenorhabditis elegans* and other nematodes, uses text mining (TM) to semi-automate its curation pipeline. In addition, WB engages its community, via an Author First Pass (AFP) system, to help recognize entities and classify data types in their recently published papers. In this paper, we present a new WB AFP system that combines TM and AFP into a single application to enhance community curation. The system employs string-searching algorithms and statistical methods (e.g. support vector machines (SVMs)) to extract biological entities and classify data types, and it presents the results to authors in a web form where they validate the extracted information, rather than enter it de novo as the previous form required. With this new system, we lessen the burden for authors, while at the same time receive valuable feedback on the performance of our TM tools. The new user interface also links out to specific structured data submission forms, e.g. for phenotype or expression pattern data, giving the authors the opportunity to contribute a more detailed curation that can be incorporated into WB with minimal curator review. Our approach is generalizable and could be applied to additional knowledgebases that would like to engage their user community in assisting with the curation. In the five months succeeding the launch of the new system, the response rate has been comparable with that of the previous AFP version, but the quality and quantity of the data received has greatly improved.

## Introduction

Biological knowledgebases are daily essential tools for researchers as they provide a point of access to multiple types of genetic and genomic data extracted from the biomedical literature. The appropriate transfer and integration of published data into these repositories relies largely on manual biocuration that, through a process of careful validation and integration, makes data easily accessible to bench scientists. Although professional digital curation of biomedical data is the best practice for sharing, managing, integrating and analyzing existing and new data, it is a costly and time-consuming process (1). Moreover, the increased volumes of digital data generated by research laboratories (2) provide a significant hurdle for databases to keep pace with published research.

To date, two main approaches to reducing the biocuration backlog have been used by biological knowledgebases. First, in ‘community curation’, authors are engaged to curate at least some of the data in their papers using web interfaces designed to facilitate data entry. Some of these efforts have been summarized in the work of Karp *et al.* (3) and to some extent have proven successful. Second, text mining (TM) approaches are used to extract entities, classify the types of data described in papers (4) and extract simple facts for curation (e.g. a mutation in gene A suppresses a mutation in gene B) (5–8). A slightly different approach to author curation has been taken by the microPublication model, which aims at having authors curate their paper during submission (9). Although the microPublication approach results in author curation at the time of data generation, it applies only to articles that are sent to the microPublication Biology journal (https://www.micropublication.org/), thus leaving out the vast majority of the research literature.

An example of a successful community curation system is that used by FlyBase (https://flybase.org/). Since 2012, FlyBase, the reference database for *Drosophila melanogaster* biology (10), has transitioned the initial triaging of papers to authors via a ‘Fast-Track Your Paper’ system (11). Authors are able to provide information on which types of data are present in the paper, provide information on newly generated antibodies and associate genes with the publication. This approach resulted in 44% of new *Drosophila*-related papers being triaged by authors. PomBase (https://pombase.org), the database for *S. chizosaccharomyces pombe*, designed a web-based tool, Canto, which allows authors to enter biological knowledge about genes, proteins and protein interactions (12). In 2015, 18% of annotations entered PomBase via Canto, saving a considerable amount of curator time. The Arabidopsis Information Resource (TAIR; https://arabidopsis.org) also allows the members of the community to submit their data using TAIR’s Online Annotation Submission Tool, with TAIR reporting high levels of precision and recall, 97% and 72%, respectively, for community-submitted, ontology-based annotations (13,14).

WormBase (WB; https://wormbase.org), the reference database for *Caenorhabditis elegans* (15) and a founding member of the recently formed Alliance of Genome Resources (https://alliancegenome.org;^16^), employs several approaches to incentivize community curation. In 2015, we implemented a community curation pipeline specifically for curating phenotypes. This pipeline regularly emails the authors of papers classified as containing mutant phenotypes and results in 10% response rate (15). In addition, since 2009, we have engaged authors, via an Author First Pass (AFP) system, to help classify data types and entities in their recently published papers. In the first AFP version ((17) hereinafter referred to as the ‘old’ AFP), corresponding authors were contacted via email shortly after their publication was incorporated into the WB curation database. The email message contained a link to a form where authors could enter lists of relevant entities and classify the data types in their paper and, optionally, provide experimental details. The form presented a simple list of data types curated at WB, followed by free-text fields where authors could include comments (Supplementary Figure S1). However, the user interface did not attempt to pre-populate entity lists using curated entity lists from WB or use autocomplete to ensure accurate data entry.

From 2009 until 2011, we received an encouraging author response rate of 40% but this rate steadily started to drop, reaching 18% in 2017 and 2018. Although the old AFP form was intuitive and simple to use and the response rate was sufficiently high, the authors needed to provide all the information as free text, a time-consuming and error-prone process. To enhance author participation, facilitate data acquisition and add controls on the type of data that can be submitted through the AFP form, we designed a completely new AFP system that takes advantage of TM approaches to aid curation. In particular, the new AFP combines some of the TM techniques already part of the WB curation pipeline, such as string-searching algorithms (18), support vector machine (SVM) (4, 19) and some of the entity extraction services provided by TextpressoCentral (https://textpressocentral.org; 6). The objective of the new AFP is to present the results of these TM methods to authors in a web-based form and allow them to validate the results of machine-based pipelines, rather than enter all information manually. In addition, the new AFP includes autocomplete functions whenever the user has to manually add data not extracted or wrongly identified by the system. This approach benefits the authors and the databases synergistically. Based on a detailed analysis of the data collected over the past 10 years with the existing AFP system, authors were reluctant to enter long lists of genes, alleles and other entities. For example, in a paper that mentions over 20 genes, authors were inclined to type in only a few gene names that they thought were most crucial to the study, leaving out other genes analyzed. By presenting them with a list of pre-populated entries, we hope to lower the barrier for participation and increase the accuracy of the submitted data.

**Figure 1:**
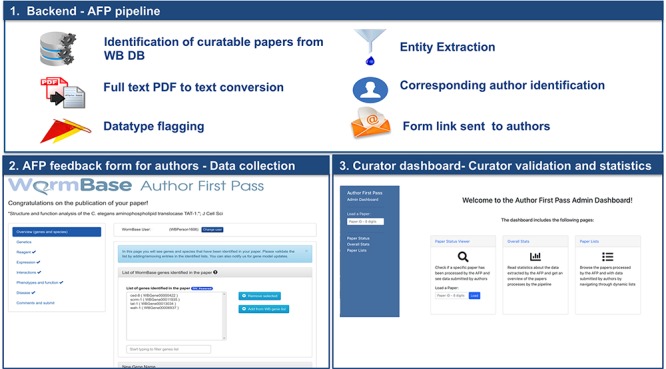
Overview of the AFP system.

From the analysis of the data collected during the first 5 months of operation, the new AFP system greatly improved the quality of curated data received from the community. In the new system, we included many more data types, ultimately providing more comprehensive data to WB. In addition, the new system includes a new interface for curators through which they can monitor the data submitted by authors and receive notifications when submissions contain data types relevant for their curation task.

## Materials and Methods

### Background on WB curation workflow: paper acquisition

The WB curation workflow starts with the identification and subsequent download, from PubMed, of the bibliographic information from potentially relevant papers. An automated script performs daily searches on PubMed looking for new papers containing the keyword ‘elegans’ in the title or abstract and previously published papers indexed with the MeSH term ‘*Caenorhabditis elegans*’. The results of these searches, specifically the paper abstract, title, journal and author list, are presented to a curator in a web-based form for manual approval. All papers that contain any experimental data on *C. elegans* are approved and, for the purposes of subsequent triage, are manually classified as ‘primary’. Papers lacking experimental data, e.g. reviews and commentaries, are manually approved based on the extent of *C. elegans*-relevant content and are manually classified as ‘not primary’. WB adds an average of 1200 papers per year to its bibliography. The full text and supplemental data of all approved publications are manually downloaded from journal websites to thus provide the input for both TM (‘primary’ and ‘not primary’) and curation (only ‘primary’) at WB (19).

### Introduction to the new AFP and overview of core functionalities

The new WB AFP system is organized into three main components (Figure 1): (i) the backend software that periodically retrieves papers from the WB internal PDF repository, converts the PDF to text, extracts and classifies relevant information and sends notifications to corresponding authors; (ii) the AFP form, which presents information extracted from papers to the authors through a web-based user interface; and (iii) the AFP curator dashboard, which allows WB curators to compare extracted and submitted data and to monitor the status of the AFP curation process through descriptive statistics on full or partial data submissions. The three components are included in a single open source repository available on GitHub at https://github.com/WormBase/author-first-pass.

**Table 1:** Data types automatically classified by the WB SVM-based paper classification system

Automatically classified data types in the AFP pipeline
• Allele sequence change
• Anatomic expression
• Genetic interactions
• Physical interactions
• Regulatory interactions
• Allele phenotype
• RNAi phenotype
• Transgene overexpression phenotype

RNAi indicates RNA interference.

**Table 2:** Entities automatically extracted by the AFP pipeline

Entity type	Example	Threshold	Special rules
Genes (including protein and sequence names)	*lin-3*; LIN-3; F36H1.4	2	1. Matched as original keyword or uppercase (proteins)
			2. A single mention in the title is considered a true positive.
Alleles	*n1059*; *sy53*; *s1263*	2	A single mention in the title is considered a true positive.
Strains	CB1417, MT1348	1	
Transgenes	*syIs107*; *zhEx68*	1	
Species	*C. elegans*, *D. rerio*, *D. melanogaster*	10	1. Additional aliases for some species (e.g. human, zebrafish)
			2. A single mention in the title is considered a true positive.

### Backend

The AFP pipeline is the backend of the AFP system and contains its core TM functions. It is executed every week, and during each run processes up to 50 newly published ‘primary’ articles (as defined above) obtained from the WB PDF repository. The identified papers are then converted to plain text through a dedicated PDF-to-text conversion module. This module combines the abstract and full text of each paper into a single plain text document. Documents with empty text, i.e. papers for which the PDF to text conversion module failed (6.5%), are excluded from the pipeline, while all others are passed to the next four steps of the pipeline: (i) binary data type classification, (ii) entity lists extraction, (iii) email address extraction and (iv) author notification.

### Binary data type classification

Papers that were successfully converted to text are passed to the external Textpresso paper classification pipeline (4), which consists of a series of SVM binary classifiers used to determine whether a paper is relevant for curation with respect to specific data types (Table 1) based on textual content. The papers are classified positive or negative for each data type by the AFP pipeline depending on the result of the SVMs. Classified papers are then sent to the next step of the pipeline, including those that were classified negative for all data types.

### Entity list extraction

In addition to classifying papers, the AFP pipeline extracts lists of entities based on WB controlled vocabularies. Given the nomenclature standards established by the *C. elegans* community (20), we are able to use regular expressions to find entity mentions in the text. Recognized entities are dynamically fetched from the WB database and include a full list of *C. elegans* genes (including sequence names), alleles, strains and transgenes (Table 2). Species names are also extracted, using a subset of species selected by WB and curated by the NCBI Taxonomy Database (https://www.ncbi.nlm.nih.gov/taxonomy).

As entities can be mentioned in papers for which they are not the main subject of experimental study, we set different thresholds for each data type to minimize the false positives, i.e. we extract entities only if they are mentioned in the document at—or above—a certain number of times. The thresholds were empirically determined by manually inspecting the full text of 40 randomly selected papers among those identified by the AFP pipeline and by setting the values so as to retrieve relevant entities while avoiding false positives as much as possible. The values of the thresholds identified with this method are reported in Table 2.

Gene names are matched both with the original keywords coming from WB vocabularies as well as by transforming them to uppercase, to match protein name mentions in accordance with the *C. elegans* nomenclature.

We extract species if we find matches to their name according to the binomial nomenclature, with the genus written in full (e.g. *Drosophila melanogaster*) or abbreviated (*D. melanogaster*). In addition, for some species, the pipeline matches additional aliases, such as ‘zebrafish’ for *Danio rerio* or ‘fruitfly’ and ‘fruitflies’ for *D. melanogaster*, as those generic terms are commonly used in research. Examples of non-relevant species mentions include anti-mouse or anti-rabbit antibodies in the Materials and Methods section or the mention of human or mouse in the Introduction or Discussion of a *C. elegans* paper for comparative, or orthology, purposes. In preliminary analyses, we observed that the main species studied in a paper are typically mentioned multiple times throughout the text, while species that are not the main focus of the paper tend to be mentioned only a few times. For this reason, the threshold for species is sensibly higher than the ones set for other data types.

For all entity types, a single mention in the title is considered a true positive by the pipeline as the information reported in the title is a faithful indication of the topic described in the paper.

After extracting the list of entities, the pipeline discards papers with no genes, alleles, transgenes or strains, as papers without entities, while included in the WB bibliography, are not relevant for the purposes of the AFP system as they likely describe experiments currently not curated by WB.

### Email addresses extraction and author contact

After entities are extracted from the text, we identify email addresses using a regular expression. The retrieved addresses are matched against curated data in the WB person class, which stores information about authors, such as name and affiliation. The first email address in the text of the paper associated with a person ID in WB is identified as ‘the corresponding author’.

The pipeline stores all the information extracted from the paper in the WB curation database and generates a link to the AFP web form. The link is emailed out to the corresponding author and contains a randomized token shared only between the author and curators at WB.

If the author does not finalize the data submission, the pipeline sends them reminder emails after 1 and 2 months, respectively. We introduced these timed repeats in August 2019, and since then, the overall response rate increased by ~5%. We also noted that many authors tend to submit data shortly after they receive the reminders.

### Frontend

#### Feedback form for authors

The AFP feedback form for authors is a web-based semi-automated tool for community curation, written in Javascript using the React framework (https://reactjs.org/). Its main purpose is to present to authors the information extracted by the AFP pipeline and to allow them to easily validate or modify the data presented.

Once a paper is processed by the AFP pipeline and the corresponding author receives a notification email with a link and the authentication token, the AFP form is ready to display the information extracted from the paper. The form first presents a welcome message with instructions on how to curate the extracted data and submit them to WB.

**Figure 2:**
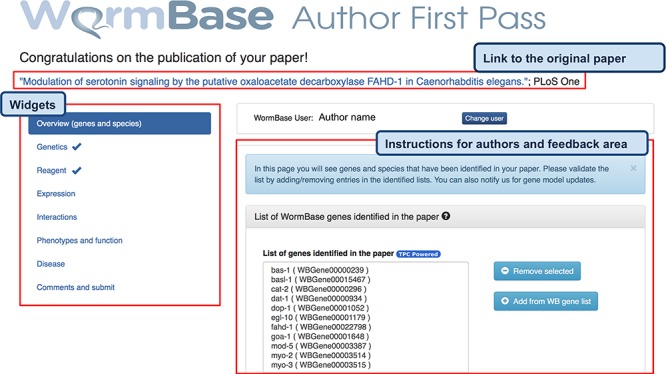
AFP feedback form for authors - Overview (genes and species) widget. The form is divided into widgets that group the data types into different categories to simplify the work for authors—left sidebar. Between the title and the main author feedback area, there is a component that displays the WormBase person name and ID for the identified corresponding author and allows the users to select a different person using an autocomplete on WormBase database. The selected person ID is stored with the curated data in our database when the submission process is completed. Each widget shows a colored panel at the top with specific instructions on how to curate the included data types. An example of the list of genes extracted can be seen in the feedback area at the center of the page.

After dismissing the welcome message, the author lands on the main page, which displays the following: (i) the title of the paper hyperlinked to PubMed; (ii) the main display area at the center, with information extracted from the paper grouped according to data types into different sections (referred to as ‘widgets’); and (iii) a left sidebar that helps navigate through the different widgets. The main layout can be seen in Figure 2.

**Figure 3:**
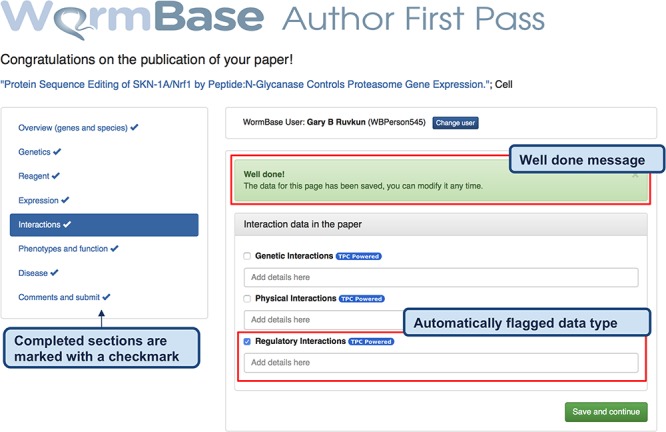
Feedback form for authors - completed form. When the author clicks ‘Save and continue’ (or ‘Finish and submit’ on the last widget), a pop-up message notifies that the data have been received by WB and the instruction alert turns green. The form returns a ‘Well done!’ message upon the completion of each section and the data are immediately stored in the WB database. In addition, to track the progress throughout the author curation process, completed sections are marked on the left menu with a special icon. The authors can modify the submitted data any time by returning to the form. The figure also shows an example of automatically classified data types, in this case regulatory interactions.

The information extracted from papers is organized in four categories, which are presented with different visual components in the AFP form. Data extracted via Textpresso Central (TPC) is labeled with a ‘TPC powered’ icon (Figures 2 and 3).

‘Automatically extracted lists of entities’ - These are presented with a select form that displays entities on different lines, as presented in Figure 2 for genes. Authors have several options to view and edit entity lists. They can filter the list to find specific entities, remove incorrectly identified entities (false positives) and add entities that were missed (false negatives) using autocompletes linked to the WB database.

‘Automatically classified data types’ - Each paper is classified by the eight SVMs presented above. The classification results are presented as checkboxes that are pre-checked if the paper is classified ‘positive’. The author can modify the status of the checkboxes (i.e. check if a false negative; uncheck if a false positive) and provide additional details in a free text input field placed below each checkbox. Author feedback will be used to retrain the SVM classifiers and improve the precision of our TM algorithms, as needed (Table 4).

‘Manually classified data types’ - These represent additional data types that are not extracted by the pipeline that are displayed as a binary value (true/false), each with a checkbox that is unchecked by default. These data types include ‘gene model correction/update’, ‘newly generated antibodies’, ‘site of action’, ‘time of action’, ‘RNAseq data’, ‘chemically induced phenotype’, ‘environmentally induced phenotype’, ‘enzymatic activity’ and ‘human disease model’. Authors can manually classify these data types via the checkboxes and can enter additional details in the associated free text box.

‘Manual entities lists’ - These are additional entities that can be submitted by the authors, such as ‘new alleles’, ‘new strains’, ‘new transgenes’ and ‘other antibodies’ used in the paper. These lists can be edited by the authors through dynamic tables, whose rows represent new entities that can be added or removed.

The form also contains links to external WB submission pages for certain type of data (e.g. phenotype data; allele-sequence details, gene-sequence details; and a link to microPublication to solicit submission of unpublished data not included in the paper). Lastly, additional comments and feedback can be submitted in a free text form on the ‘Comments and Submit’ widget.

After the author has reviewed or modified the data presented on each page, the information is stored directly in the WB curation database. For example, genes and species, two entity types directly associated with papers in the curation database, are automatically entered into the appropriate database tables with evidence indicating the associations were made via the AFP system. Lastly, an email is sent to a list of WB curators to notify them of the new submission.

#### ‘My AFP Papers’ page

**Figure 4:**
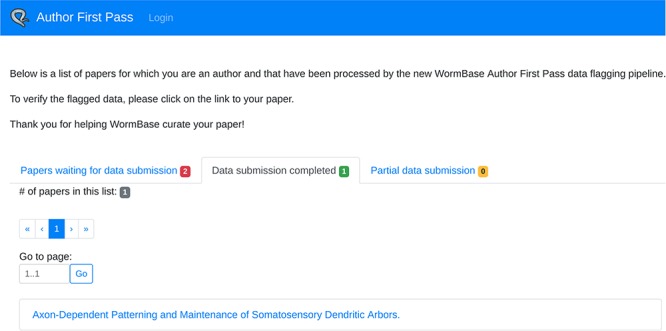
‘My AFP papers’ page. The authors can retrieve links to the AFP feedback form for their papers through the interface presented in this page.

In addition to being able to access a specific paper via the link sent in the email, authors can retrieve the complete list of their papers processed by the AFP pipeline (for which they are authors, either corresponding or not) via the ‘My AFP Papers’ page (www.textpressocentral.org:5002). The page is divided into three tabs (Figure 4): (i) papers waiting for data submission, (ii) papers for which the data submission is completed and (iii) partial data submissions.

#### Curator dashboard

**Figure 5:**
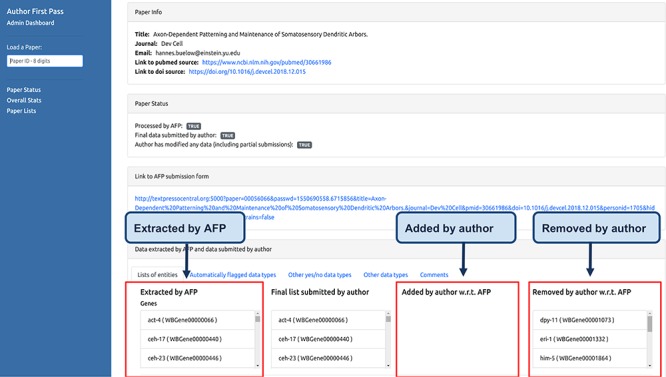
Curator dashboard. Curators at WB use this page to monitor the AFP system and to compare the data extracted by the pipeline with those submitted by the authors.

The AFP curator dashboard is a web application that allows WB curators to monitor the status of the AFP curation process. It is divided into three pages. The first page (Figure 5) can be used to check if a specific paper has been processed by the AFP pipeline and allows visualization of the differences between the data extracted by the pipeline and the data submitted by the authors.

**Figure 6:**
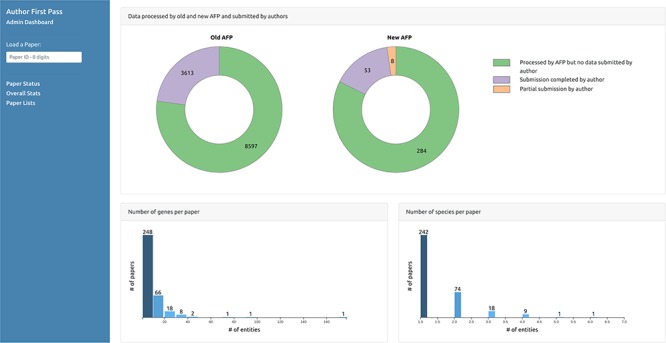
Curator dashboard - Statistics page. Curators can collect statistics on the extracted and submitted data through this page.

**Figure 7:**
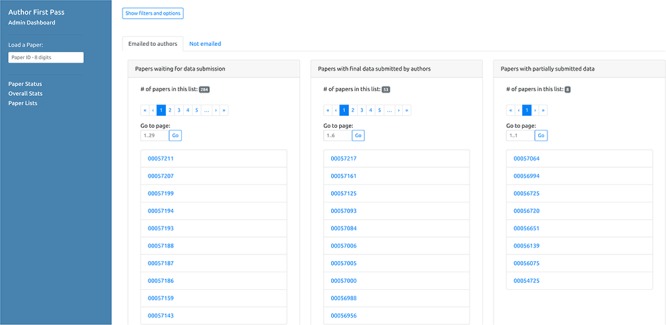
Curator dashboard - Paper lists. This page of the curator dashboard contains links to the feedback form for authors for all papers processed by the pipeline, divided into different groups depending on their status (i.e. processed but not submitted, full submissions and partial submissions).

The second page (Figure 6) displays graphs of the overall status of the AFP curation process, reporting, for example, the total number of papers processed by the pipeline and the number of submissions received from the authors, including partial submissions. It also displays graphs depicting distributions of the number of entities automatically extracted from the papers for each data type. The third page (Figure 7) displays the lists of paper IDs processed by the pipeline and those with data submitted by the authors. Each PaperID is hyperlinked to the page that shows the status of a specific paper and can be used by the curators to easily verify the data extracted by the pipeline and the quality of data submitted by the authors. The lists can be filtered by data type to allow curators to retrieve relevant submissions with respect to their curation tasks. Access to the AFP dashboard is restricted to WB curators.

## Results

### User response analytics

To evaluate the advantages of the new AFP system, and to monitor the quantity and quality of the data submitted by the authors, we compared the data processed by the old and new AFP systems, including the author’s feedback. In particular, we analyzed the number of papers processed by the old system over the years and the response rate from authors collected from April 2009 until May 2019 (when the old system was replaced by the new one). We then compared these numbers with the results acquired from the first months of service of the new AFP, which was tested from March 2019 to May 2019 and officially released in June 2019. We performed a detailed analysis on the accuracy of the data extracted by the new AFP system, comparing it with the data submitted by the authors, to assess the capacity of the system to provide meaningful suggestions with the pre-populated fields. Finally, we provide details for the additional data received through the form for non-pre-populated fields.

### Previous AFP system

The first AFP version was released in April 2009. From 2009 to 2018, the system processed papers at an average pace of approximately 1000 papers per year (with an average of 82.7 ± 45.9 papers processed per month), for a total of 12 208 papers processed in 11 years. The maximum number of papers processed by the old AFP system per week was set to 50, which allowed us to remain within the limits imposed by the email service provider. The system hit the cap of 50 papers per week in June 2009 but remained otherwise under the threshold, meaning that the cap did not limit the number of papers that were processed by the AFP. This allowed us to cover all the new *C. elegans* articles published per week. We therefore kept the same value with the new AFP system.

**Figure 8:**
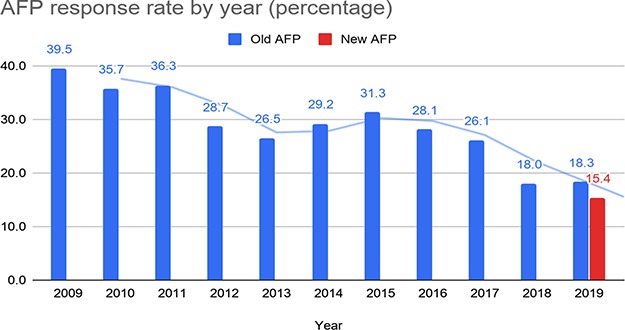
Response rate for the old and the new AFP.

The submissions received by the authors for the old AFP were approximately 300 per year between 2009 and 2019, for a total of 3612 submissions. Figure 8 depicts the percentage of submissions received with respect to the number of papers processed by the old system (blue bars for each year in the chart). The solid lines in the chart represent the running average calculated over two data points for each set of bars. The response rate for the old AFP gradually declined over the years, from 39.5% in 2009 to 18% in 2018 and 18.3% in the first part of 2019.

**Figure 9:**
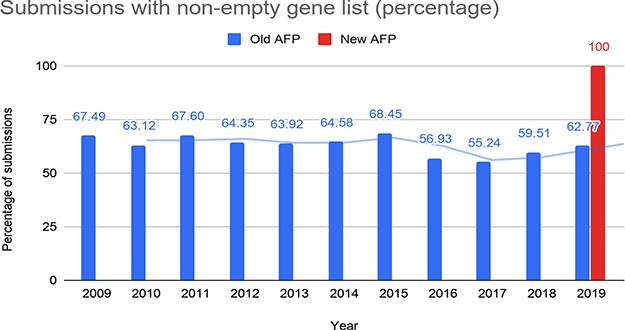
Percentage of submissions with at least one gene reported through the old and the new AFP.

#### New AFP system

As of 02 October 2019, the new AFP system processed and sent out emails for 345 papers. A total of 48 papers (i.e. 12.21% of the total number of papers processed) were discarded by the pipeline because they did not contain any genes, alleles, transgenes and strains and were therefore not relevant for the AFP curation. No emails were sent out for these papers, but they can be retrieved in the curator dashboard for testing purposes. Those 48 ‘empty’ papers are excluded from the analysis below, which focuses on papers for which an email was sent to the authors.

We received 53 complete submissions and 8 partial submissions. The percentage of the complete submissions received so far (15.4%) is only slightly lower than the percentage of the submissions received for the old AFP in the first part of 2019 (18.3%). Nonetheless, we observed that the results collected through the new system are far more complete and accurate than the old one. Since the old AFP feedback page was a collection of free-text fields, there was no control on the completeness and accuracy of the data submitted by authors, especially for data types that required more effort by the author, such as a long list of genes studied in the paper. The overall response rate recorded for the old AFP may thus be higher than the response rate of complete submissions, especially for gene lists. In addition, the old system sent out emails for papers without entities, and these papers may have increased the response rate for the old form as it was easier for authors to provide feedback for papers with no entities.

#### Response density (number of entities provided in the submissions)

We calculated the percentage of submissions with non-empty gene lists for the old and the new AFP over time (Figure 9). In the old AFP roughly two-thirds of the submissions contained at least one gene, whereas the submissions for the new AFP all contain non-empty gene lists.

**Figure 10:**
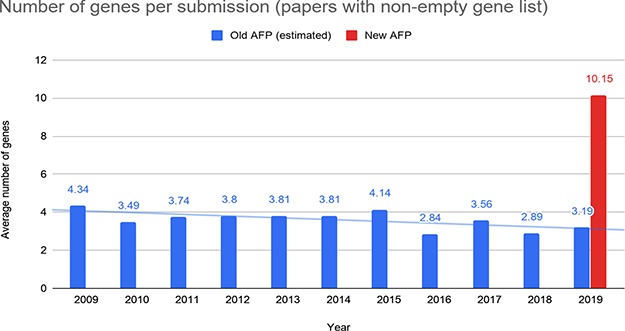
Number of genes reported by the authors per submission through the old and the new AFP forms.

To better understand the data submitted through the forms, we further analyzed all the submissions where at least one gene was reported. The average number of genes provided through the old form was significantly lower than the one provided through the new form (roughly 2- or 3-fold lower; Figure 10). This further confirms that the submissions provided through the new AFP are more comprehensive than those coming from the old system.

#### Accuracy of pre-populated data based on author’s feedback

To calculate the accuracy of the automatic entity extraction process, we compared the data extracted from the AFP pipeline and the data submitted by the authors through the form. We calculated the Jaccard coefficient (a standard similarity measure for pairs of sets that goes from 0 when two sets are disjunct to 1 when two sets contain the same elements) for the lists of entities extracted by the pipeline, i.e. genes, species, alleles, strains and transgenes. The results, reported in Table 3, show that we were able to extract entities with high accuracy (~86% on average). In particular, the accuracy is high for genes, alleles and strains; lowest in species; and very high for transgenes. We noted that, in many cases, the low accuracy for species is due to PDF to text conversion errors. As we explain in more detail in the Discussion section, we plan to improve our PDF to text conversion pipeline by adding custom functions specifically designed for the extraction of our data types.

**Table 3:** Accuracy of the automatic entity extraction process, based on author response

Data type	AVG Jaccard coefficient	AVG number of entities added per submission	AVG number of entities removed per submission
genes	0.85	0.38	1.23
species	0.75	0.28	0.36
alleles	0.88	0.26	0.43
strains	0.88	0.25	0.40
transgenes	0.95	0.04	0.09

The Jaccard coefficient measures the similarity between the set of automatically extracted entities and the set of entities submitted by authors through the feedback form.

AVG indicates average.

Table 3 also contains the average number of entities added or removed per paper by authors (columns 3 and 4, respectively). From the results in the table, we note that authors tend to remove more entities than the ones they add, and, in general, the number of changes they make to the extracted data is low—less than one entity removed and added per paper, apart from removed genes, which average slightly above one.

We further analyzed the list of entities removed by the authors, to better understand how we could decrease the rate of false positives (see Discussion). Overall, 29 unique genes were removed. In the majority of cases, genes were removed either because they were not the main focus of the study (*n* = 10) or because they were used as reagents, i.e. 3′UTRs (untranslated regions), control transgenes and coinjection markers (*n* = 12). Two genes were correctly removed by the authors pointing out a bug in our synonym-matching algorithm. In three instances, the authors incorrectly removed genes that were experimentally studied. Authors removed allele and strains either because they were not the main focus of the study or because the string matching identified false positives. For example, ‘*m2*’, a bona fide *C. elegans* allele, matched the symbol for ‘square meters’. Only three transgenes were removed by the authors, as they were used as controls.

For genes and species, we also manually validated the submissions received, providing an in-depth comparison between the curation data provided by the authors and the curation performed by a WB curator on the same papers. This validation was performed on a sample of 10 articles randomly selected from the submissions received through the AFP system. We found that author-provided gene lists have a 61.8% precision and 82.3% recall with respect to the list of genes extracted by a curator and species have 100% precision and recall. This means that the list of genes and species received from the authors are in line with the WB curation, even though some of the genes submitted by the authors are not relevant for curation, as witnessed by the medium value for precision (see Discussion). Note that even if we account for false positive genes with respect to those curated by WB in Figure 10, we still have, on average, about 6 out of approximately 10 genes submitted by the authors that are true positive, still higher than the average number of genes submitted through the old AFP.

#### Accuracy of the SVM-based classification based on author’s feedback

For the binary data types pre-populated by the WB SVM-based paper classification pipeline, we calculated the accuracy (percentage of correct positive or negative predictions over the total number of submissions received), precision and recall of the predictions made by the SVMs compared with the results submitted by the authors. This is reported in Table 4 for all data types that are associated to an SVM, along with the number of papers that were classified positive for each data type by the respective SVM.

**Table 4:** Accuracy, precision and recall of the SVM-based classification calculated by comparing the SVM values and the values submitted by the authors considering all submissions received

Data type	Author–SVM agreement accuracy	Author–SVM agreement precision	Author–SVM agreement recall	Number of papers classified
Allele sequence change	0.94	0.90	0.95	20
Anatomic expression	0.87	0.83	0.67	15
Genetic interactions	0.89	0.75	0.75	12
Physical interactions	0.94	0.8	0.67	6
Regulatory interactions	0.81	0.77	0.59	17
Allele phenotype	0.85	0.96	0.77	31
RNAi phenotype	0.92	0.83	1.00	19
Transgene overexpression phenotype	1.00	1.00	1.00	13

**Table 5:** Accuracy, precision and recall of the author-based classification calculated by comparing the values submitted by the authors and the curation performed by WB curators on the submissions received

Data type	Author–curator agreement accuracy	Author–curator agreement precision	Author–curator agreement recall
Anatomic expression	0.87	0.87	0.72
Genetic interactions	0.76	0.83	0.48
Physical interactions	0.96	0.83	0.83
Regulatory interactions	0.77	1	0.59
RNAi phenotype	0.89	1	0.76

The accuracy for the data types pre-populated by SVMs is quite high, with an average of ~90%, a minimum value of 81% for ‘regulatory interactions’ and a maximum of 100% for ‘transgene overexpression phenotype’. This means that the suggestions made by the pipeline on the processed papers were accurate and the authors made relatively few changes to the extracted data. This is also reflected in the high precision, whereas the recall is high for most data types but medium for ‘anatomic expression’, ‘physical interactions’ and ‘regulatory interactions’.

It is worth noting that the results obtained here by the SVMs with respect to the values submitted by the authors are in line with the evaluation results previously obtained on larger test sets (4).

To evaluate if the authors classified the papers correctly, WB curators validated a subset of classified data types for the 53 papers for which we received submissions (Table 5). The values indicate that the submissions received by the authors are accurate, even though the recall is low for ‘genetic interactions’ and ‘regulatory interactions’, which are data types known to be particularly hard to classify with SVMs. We will work on improving the results by better communicating to authors the type of experimental results relevant for WB curation.

#### Manual list of entities submitted by the authors

The form allows the authors to submit new entities, i.e. alleles, strains, transgenes and antibodies, that are not yet present in the WB database. The average number of entities manually submitted by the authors per paper is shown in Table 6.

**Table 6:** Average number of non-pre-populated entities submitted by the authors

Data type	Average number of entities
New alleles	0.89
New strains	3.58
New transgenes	0.51
New antibodies	0.28

In addition, we allow the authors to classify additional data types, which are curated at WB but do not have an SVM classifier (Table 7). The list of new entities and of the additional data types classified is sent to relevant curators for inclusion in the WB database via manual curation.

**Table 7:** Percentage of papers classified for specific data types for which no automated suggestions were provided and comparison between author classification and WB curator classification

Data type	Percentage of classified submissions	Author–curator Accuracy	Author–curator precision	Author–curator recall
Gene model correction/update	1.89%			
New antibody	3.77%			
Site of action	13.21%	0.88	0.5	0.5
Time of action	5.66%			
RNAseq data	5.66%			
Additional type of expression data	15.09%	0.96	0.88	0.88
Chemical induced phenotype	5.66%			
Environmental induced phenotype	9.43%			
Enzymatic activity	3.77%	0.96	0.5	0.5
Human disease model	33.96%	0.87	0.67	0.92

Partial submissions are not counted in the table.

Lastly, the authors provided comments via the free text box at the end of the feedback form for 15.09% of the submissions. In some cases, the authors have highlighted specific aspects of their papers in the comments and, in one case, sought clarification on the transgene entity, which we have attempted to rectify by adding another example to the data entry field.

For some of the data types in Table 7, we performed an additional validation by comparing the data provided by the authors with the data curated by WB curators on the full set of 53 papers submitted through the new AFP. The accuracy, precision and recall of the authors’ submissions with respect to WB curation are reported in columns 3–5 in the table. The results of this author–curator comparison show that the manual classification done by the authors is accurate and precision and recall are high for certain data types (‘additional type of expression data’ and ‘human disease model’). The medium/low values of precision and recall for ‘site of action’ and ‘enzymatic activity’ tells us that we need to better communicate the definition of these data types to the authors, as in some cases the classification is not in line with what WB curators would expect.

## Discussion

Only five months after going to production, the new AFP system has already provided us with valuable information about author participation in our curation process. The quality of author submissions has improved compared with the old AFP system, as witnessed by the data analysis reported in this paper. The introduction of the new AFP system also improved the curation efficiency. Specifically, the authors are classifying data types for which the SVM results were incorrect or for which we do not have an SVM at all (e.g. human disease model). Since WB curators do not routinely review SVM negative papers as this would be a manual, labor-intensive process, the AFP pipeline saves time by alerting us to papers and data types that might otherwise go uncurated. For entities, we note specifically that author-verified gene submissions, while still not as precise as we would like, result in greater than 5-fold increase in the number of genes associated with papers in our curation database. In addition, for species associated with papers in WB, we note that the AFP pipeline results in 100% precision and recall of author-submitted data with respect to WB curation, decreasing the curators’ effort. In particular, we find that author-verified species help clarify, for example, species otherwise referred to in paper abstracts as ‘mammalian’ or ‘bacterial’, a clarification that would require a curator to manually review the paper. Lastly, the comments that the authors enter in the free text box next to the data type classification greatly facilitate curation as they direct curators to the relevant information that needs to be extracted.

From the 53 completed submissions we have received to date, we also analyzed several aspects of author participation, as discussed below.

### Analysis of entities validated by the authors

For entity lists, we have begun to see trends in what entries the authors remove, which is likely indicative of false positives from the perspective of what is deemed significant for their research findings rather than false positives based on incorrect string matching. For example, some genes’ promoters are commonly used for tissue-specific gene rescue experiments to evaluate the potential site-of-action of a given gene. In many—but not all—cases, the authors removed these genes from the gene entity list as we might have expected. Based on the fact that not all authors removed these genes, even when they were employed similarly, we will explore improving the system by (i) communicating better to the authors that we are interested in capturing genes with significant experimental findings; (ii) developing a ‘blacklist’ for genes that we know are likely to be used as experimental reagents, where the blacklisted genes will still be presented to the authors but marked as possible reagents; (iii) using a higher threshold for the extraction; and (iv) employing other methods instead of thresholds such as term frequency–inverse document frequency, a numerical statistic that is intended to reflect how important a word is to a document in a collection or corpus. We may also employ more sophisticated methods, such as comparing the frequency of gene mentions in the *C. elegans* TextpressoCentral corpus with the manually curated gene-paper associations previously performed at WB, to generate a candidate ‘blacklist’; those genes with a low ratio of WB:TextpressoCentral mentions would be top candidates.

The analysis of entity addition and removal has also given us insight into how we might better present information to improve the accuracy of author validation. Our AFP form currently lists different entities in separate sections of the form, e.g. gene lists are in a separate box from allele lists. In reality, though, genes and alleles are linked to one another as well as to strains that refer to specific genotypes. We found a number of cases where authors removed a gene from the entity list but not the corresponding allele. Although we are not sure why the authors did this (perhaps gene names are more easily recognized than allele names), in future versions of the form we plan to link genes, alleles and strains so that when the authors remove one of the entities, they are notified of the presence of the linked entities. Presenting entities in this ‘linked’ manner will require further processing of extracted data but could use existing curated WB data to provide the appropriate mappings.

Another possible strategy to avoid false positives would be to make sure that if, for example, an allele is extracted, the corresponding gene is extracted as well. For instance, in the case of the *m2* allele false positive, its corresponding gene, *unc-26*, was not mentioned in the paper, an indication that *m2* may not refer to a *unc-26* allele name. Similarly to the blacklisted genes, these alleles can be shown to the authors marked as potential false positives.

### Analysis of data types validated by the authors

In general, automatically classified data types are in excellent agreement with author validation. This may reflect, in part, that the SVMs used to classify these data types have been in use at WB for many years and, in some cases, have undergone extensive curator testing and feedback. Thus, the accuracy of the classification is very good. For the two data types with the lowest agreement, regulatory interactions and allele phenotype, classifying papers for the particular data may inherently be difficult (especially for regulatory interactions) and further work would be needed to develop a more accurate classification method. We note that in some cases, even when the agreement is high, there may still be papers that are not positive for WB curation (e.g. chemical–gene product interactions). In these cases, we will try to provide better descriptions of the types of data we wish to classify while at the same time monitor potentially new types of data that we may wish to consider curating in the future.

Author-submitted data types are particularly valuable for our system, as these are data types for which we currently lack other means of classification. For example, site- and time-of-action studies, critical for understanding where and when a gene product functions, are not currently classified by WB, and thus author classification for these papers is essential for triaging. As with entities and classified data types, though, we note that effectively communicating what is relevant for WB curation is also important here, as the authors may incorrectly classify their papers for data types that do not meet our criteria for curation. Classification for human disease relevance is of particular note, as while the significance of using model organisms to study human disease is well documented, not all papers that cite human disease relevance can be curated within the constraints of the WB human disease data model.

### Limitations of PDFs as inputs for TM

Our TM pipeline relies on converting the documents in PDF format to text for subsequent entity recognition and data type classification. In particular, the PDF-to-text conversion module is the same used by Textpresso, and it is publicly available (https://github.com/TextpressoDevelopers/tpctools). While the PDF-to-text conversion works successfully for many articles, in some cases it fails (6.5% of the total number of processed papers—in line with the results obtained by Textpresso on a larger corpus), thus limiting our ability to process all potential papers for the AFP pipeline. The difficulties of accurately extracting text, and figures and legends, from PDFs is a known barrier to TM in the Biomedical Sciences (21,22). One possible, albeit limited, solution would be to process as many articles as possible using the PubMedXML format as source text. This format would only be available for open-access articles, but the trade-off for more accurate text processing might justify the development of this alternative text processing pipeline. From the set of articles that failed our PDF-to-text conversion (6.5% of the total), 25% are open access. Thus, we could reduce the percentage of articles that cannot be converted to ~5% if we use their open access XML version when available.

We also calculated the number of entity extraction errors due to PDF-to-text conversion issues. There were 22 entities (6 genes, 5 alleles, 6 strains, 5 species) not extracted by the pipeline due to conversion errors but added by the authors in the form, therefore false negatives. Since the seven articles from which the false negatives were identified are all open access articles (i.e. Elife, PLoS), using the PubMedXML format as source text will solve the conversion issue, at least for those publishers.

### Improving author participation

After 5 months in production, our current author participation rate is 15.4%. While this is in accordance with the participation rate of the old AFP system for 2019, and any author participation is of benefit to WB, we would like to significantly improve our response rate. Two approaches are of particular note here. The first is to recognize author participation by acknowledging them on the WB homepage and the corresponding paper page. By recognizing the authors who participate in AFP, we hope to communicate how important this is to our curation pipeline. We are also considering using ORCID for authentication to link WB curation with the author’s ORCID profile in order to acknowledge their contribution. Examples of this kind are currently in place in Reactome (https://reactome.org) and UniProt (https://www.uniprot.org). This may give an incentive to authors to contribute. The second is to directly communicate with our user community on the value of AFP and other community curation. Over the past years years, WB has given a number of workshops in geographical areas with a large concentration of *C. elegans* researchers. Such workshops, along with the biannual *C. elegans* meeting, are ideal opportunities to engage authors with hands-on tutorials on using the new AFP interface. In addition, these user interactions allow us to further educate the WB user community on what information is critical to include in publications for successful curation.

## Conclusion and future directions

Efficient and accurate literature curation is one of the primary goals of model organism databases (MODs). To improve the curation efficiency, some MODs employ community curation, largely performed by authors or TM to augment manual literature curation pipelines. In this paper, we describe a novel-combined approach that uses TM pipelines developed at WB as inputs to a new community curation platform. This combined approach shifts the focus of community curation from data input to data validation with the goal of lessening the participatory burden on community curators and improving the performance of TM algorithms.

The TM pipelines that we use identify entities, such as genes, alleles, strains and species, as well as other types of data, such as expression patterns or genetic and physical interactions, described in published papers. Information derived from these pipelines, verified by the authors, thus become the basis for WB curation prioritization, or triage and provide paper-entity associations in the WB bibliography.

Launched in June 2019, the new WB AFP system has provided not only essential WB curation but also valuable insight into how authors interact with our community curation form.

In the future, we hope to extend this system to the other MODs to help engage their user communities as well. As WB is part of the Alliance of Genome Resources, and one of the goals of the Alliance is to harmonize the curation practices wherever possible, we foresee a potential for collaboration with the other Alliance members to generalize the system for their literature corpus. Indeed, the AFP system could be generalized to help with entity recognition and data type classification for any database that relies on literature curation and makes use of controlled vocabularies and persistent identifiers, a core component of downstream database integration (23). In addition, as new experimental techniques become more widely adopted (RNA interference is a notable past example), we will update the AFP form to allow for classification and validation of new data types. Such updates will keep the AFP system relevant and helpful for our user community and curation efforts.

## Supplementary Material

S1_baaa006
