# Supplementary figures and images for: Text mining meets community curation: a newly designed curation platform to improve author experience and participation at WormBase
Source: Database (Oxford). 2020 Mar 17;2020:baaa006. doi: 10.1093/database/baaa006 (PMC7078066; doi:10.1093/database/baaa006)

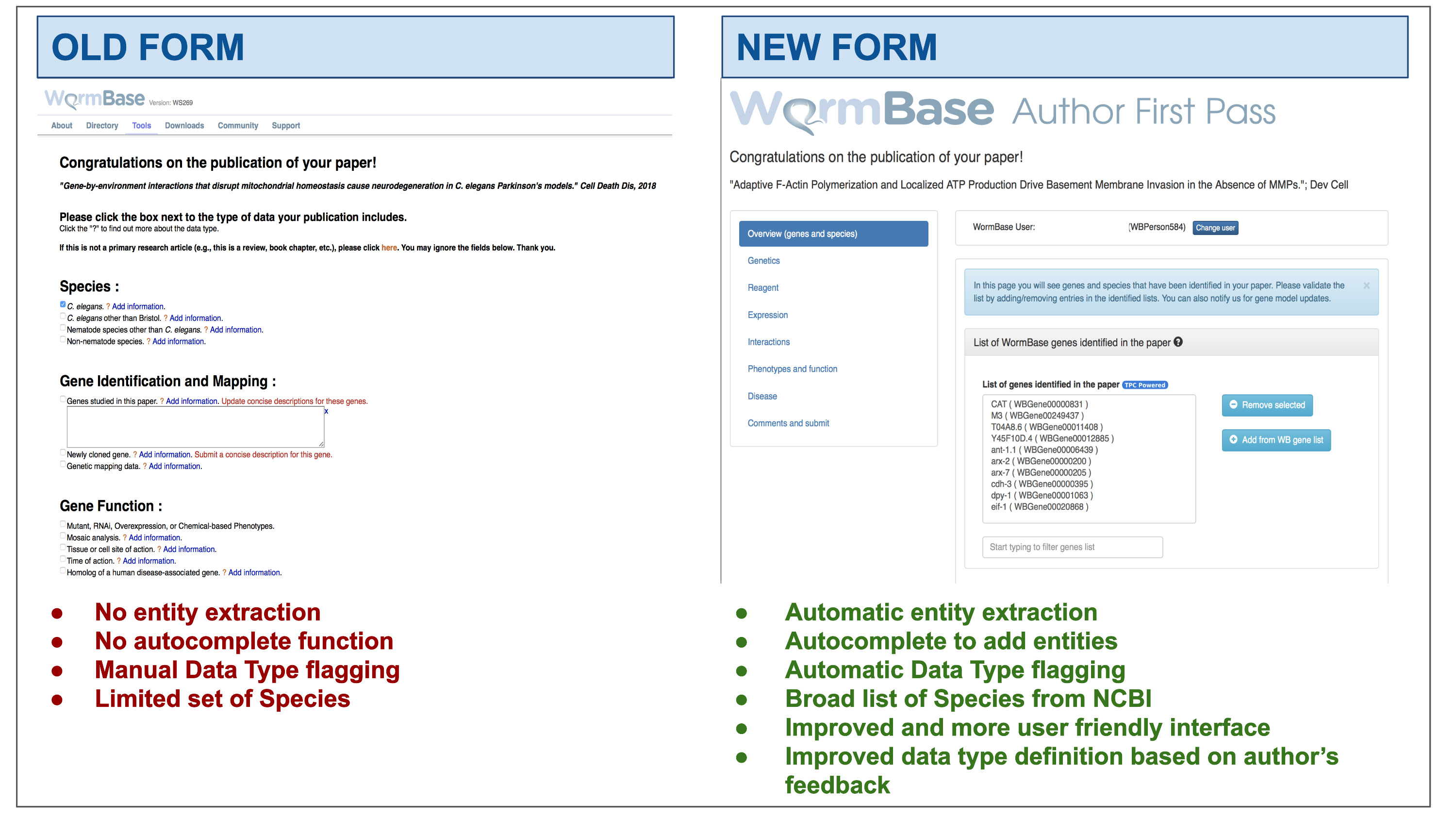

Supplement: S1_baaa006 [file s1_baaa006.jpeg]
